# Reaction of F Atoms with Carbonyl Sulfide: Experimental and Theoretical Study

**DOI:** 10.3390/molecules29215027

**Published:** 2024-10-24

**Authors:** Yuri Bedjanian, Manolis N. Romanias

**Affiliations:** 1 Institut de Combustion, Aérothermique, Réactivité et Environnement (ICARE), CNRS, 45071 Orléans, France; 2 IMT Nord Europe, Institut Mines-Télécom, Centre for Energy and Environment, University Lille, 59000 Lille, France; emmanouil.romanias@imt-nord-europe.fr

**Keywords:** fluorine atom, carbonyl sulfide, rate coefficient, temperature dependence, negative activation energy

## Abstract

The gas phase reaction of fluorine atoms with carbonyl sulfide, F + OCS → SF + CO (1), has been investigated over a wide temperature range, T = 220–960 K, in a discharge-flow system combined with mass spectrometry for the analysis of the reactive mixture. The reaction rate coefficient was determined as a function of temperature using both absolute (under pseudo-first-order conditions using an excess of F atoms) and relative rate methods: *k*_1_ = (8.95 ± 0.52) × 10^−11^ exp((102 ± 20)/T) cm^3^ molecule^−1^ s^−1^ (with an estimated total uncertainty of 15% independent of temperature). The SF radical was observed as the main primary reaction product. The experimental results are supported by theoretical calculations, which evidence that the reaction is exothermic with a weak negative temperature dependence.

## 1. Introduction

Reactions of atomic fluorine with closed-shell molecules are generally very rapid and lead to the generation of diverse active species. This specificity of the reactions of fluorine atoms determines, in addition to theoretical interest, practical interest in these reactions associated with laboratory studies of gas-phase kinetics, where the reactions of F atoms can potentially be used as a source of various atoms and radicals, as well as titration reactions for detecting and measuring the absolute concentrations of the active substances formed. The existing database on the reactions of F atoms is very limited and uncertain: the rate constants of many studied reactions of fluorine atoms are determined with insufficient precision even at room temperature, let alone the temperature dependence [1]. The main reason for this is the rapidity of the reactions (rate constants are often close to the frequency of bimolecular collisions) and the presence of highly reactive species in the reaction products, which imposes certain requirements on detection sensitivity to limit the potential impact of rapid secondary chemistry.

In the present work, we report the results of the combined experimental and theoretical study of the reaction of F atoms with carbonyl sulfide.

F + OCS → products (R1)

Reaction (R1) is a potential source of SF radicals. It can be also of potential interest for the chemistry of hot near-source volcanic plumes, where OCS and fluorinated compounds are simultaneously emitted [2]. Note that for reaction (1) only one previous study at room temperature is available [3].

The aim of this work was to experimentally determine the rate constant of the title reaction in an extended temperature range T = 220–960 K and to obtain information on the reaction mechanism through direct detection of the reaction products and theoretical thermochemical calculations.

## 2. Results and Discussion

### 2.1. Rate Constant of Reaction (R1): Absolute Measurements

**Figure 1:**
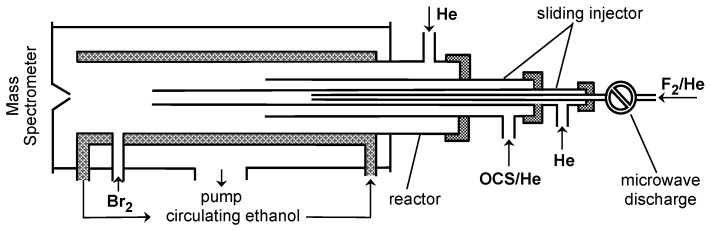
Diagram of the low temperature flow reactor: configuration used in the absolute measurements of *k*_1_.

The configuration of the flow reactor used in the absolute measurements of the rate constant of Reaction (R1) is shown in Figure 1.

**Figure 2:**
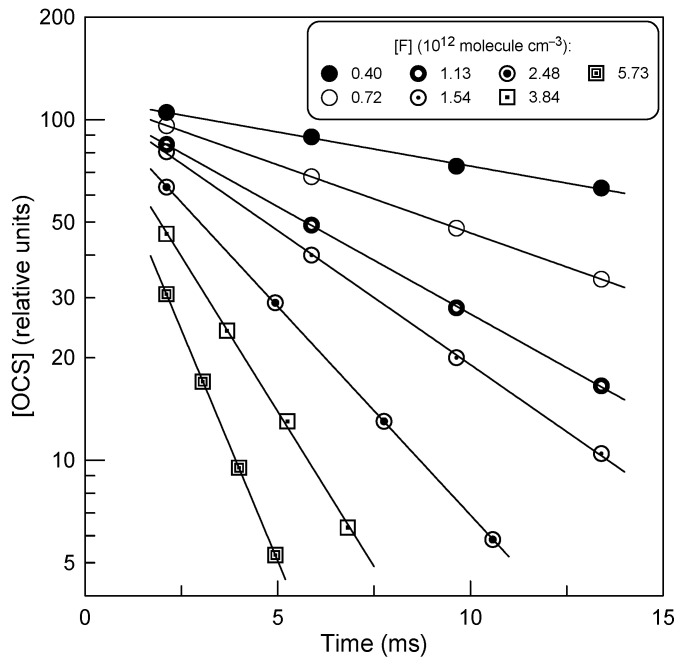
Plots of OCS concentration against reaction time for various concentrations of fluorine atom (T = 300 K).

The measurements of *k*_1_ consisted of monitoring the kinetics of the OCS (initial concentration, [OCS]_0_, of (1 − 2) × 10^11^ molecule cm^−3^) consumption under an excess concentration of F atoms ([F] = (0.35 − 6.20) × 10^12^ molecule cm^−3^). The advantages of working with excess of F atoms over OCS were as follows: (i) better detection sensitivity for OCS, allowing the use of low initial concentrations of OCS, and (ii) non-involvement of OCS in possible secondary reactions, unlike the fluorine atom. Examples of the exponential decays of OCS, [OCS] = [OCS]_0_ × exp(−*k*_1_’ × t), where *k*_1_’ = *k*_1_ × [F] is the pseudo-first-order rate constant and [F] represents the average F-atom concentration along the reaction zone, are shown in Figure 2.

**Figure 3:**
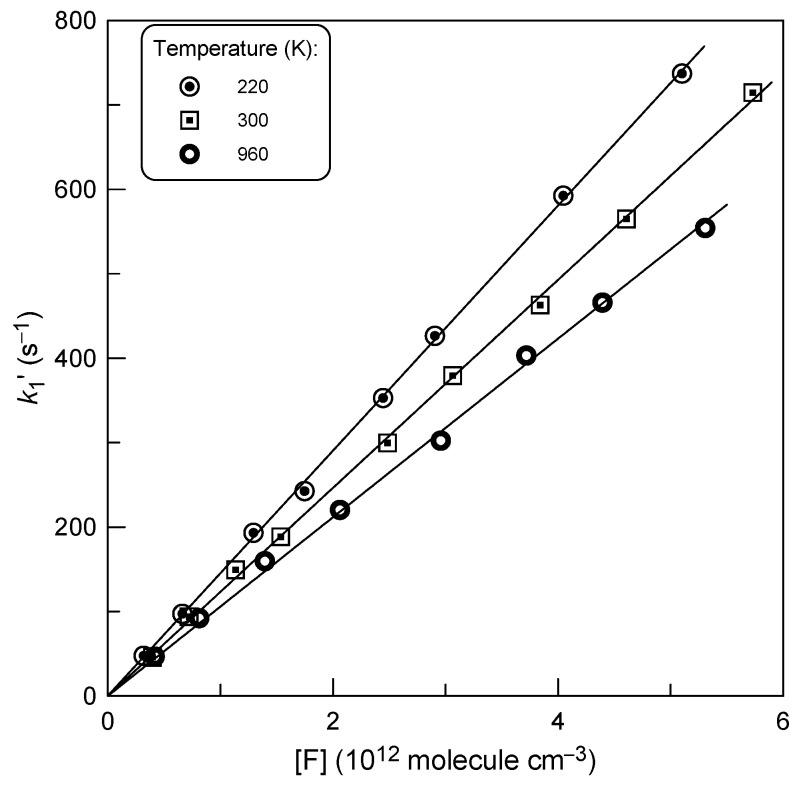
Pseudo-first-order rate constant *k*_1_′ = *k*_1_ × [F] as a function of F-atom concentration at different temperatures.

The consumption of F atoms due their heterogeneous loss (*k*_w_ ≤ 10 s^−1^) and reaction with OCS was insignificant, reaching a maximum of 20% in a few kinetic runs. The diffusion corrections applied to *k*_1_’ values, determined from the data like those in Figure 2, were generally <10% and up to 20% in a few kinetic runs. Examples of second-order plots measured at different temperatures are shown in Figure 3. The rate constants of the Reaction (R1) obtained from a linear through origin least-squares fit of the *k*_1_’ data as a function of [F] are given in Table 1. The combined uncertainty of the absolute measurements of the rate constants was estimated to be ≤15%, including (in quadrature) statistical error and those of the measurements of the flows, pressure, temperature and absolute concentration of F atoms.

### 2.2. Rate Constant of Reaction (R1): Relative Rate Measurements

**Figure 4:**
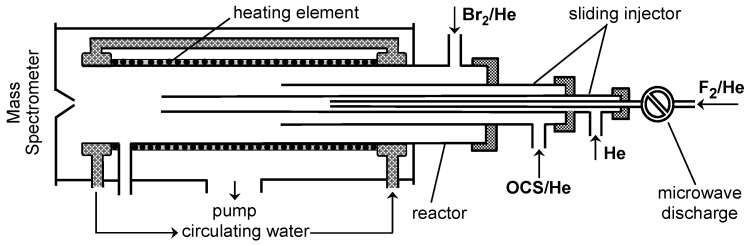
Diagram of the high temperature flow reactor: configuration used in the relative measurements of *k*_1_.

Figure 4 displays the configuration of the flow reactor used in the relative measurements of *k*_1_.

**Figure 5:**
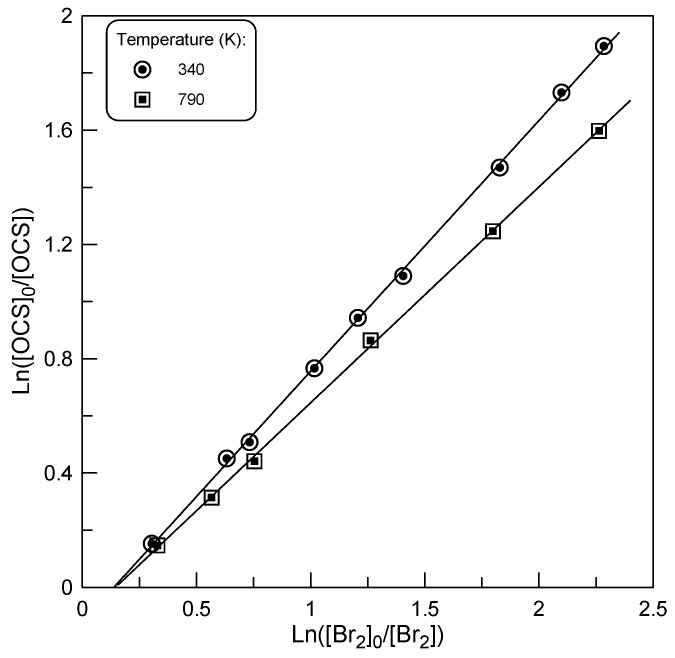
Dependence of ln([OCS]_0_/[OCS]) on ln(Br_2_]_0_/[Br_2_]) observed at T = 340 and 790 K.

In the first series of relative rate measurements, the rate constant of Reaction (R1) was determined using a relative rate method (RRM1), comparing the decay rates of OCS and reference compound, Br_2_, simultaneously present in the reactor, in reactions with F atoms (Figure 4).

(1) $$ln\frac{[OCS{]}_{0}}{\left[OCS\right]}=\frac{k_{1}}{k_{2}}\times ln\frac{[{Br}_{2}{]}_{0}}{\left[{Br}_{2}\right]}$$

where the expressions under the logarithm are the ratios of the compound concentration in the absence to that in the presence of F atoms at a given reaction time. Examples of the experimental data are shown in Figure 5.

The final values of *k*_1_ (Table 1) were determined using *k*_1_/*k*_2_, derived from the slopes of the straight lines in Figure 5, and the rate constant of the reference reaction.

F + Br_2_ → Br + FBr (R2)

where *k*_2_ = (1.28 ± 0.20) × 10^−10^ cm^3^ molecule^−1^ s^−1^ (T = 299–940 K) [4].

**Figure 6:**
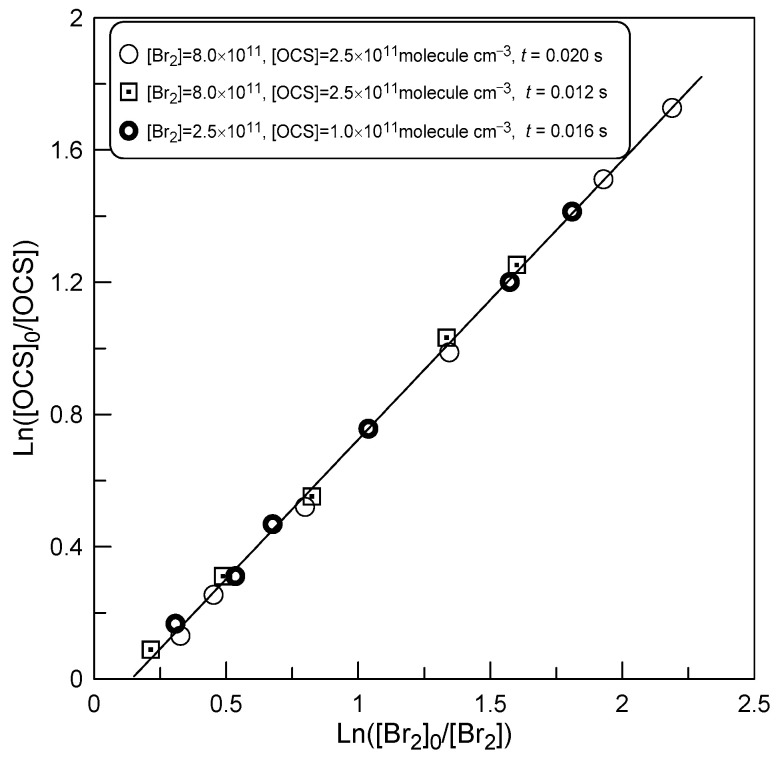
Dependence of ln([OCS]_0_/[OCS]) on ln(Br_2_]_0_/[Br_2_]) observed at T = 420 K under different experimental conditions.

Although the results of these relative measurements are in good agreement with the absolute data (Table 1), it should nevertheless be noted that the lines in Figure 5 do not pass through the origin, as one would expect from Equation (1). This seems to indicate additional consumption of Br_2_ in the side processes. In search of potential reasons for the observed effect, we conducted additional experiments at T = 420 K by varying the reaction time and initial concentrations of the reagents. The results are presented in Figure 6.

One can note that variation in the reaction time (by a factor of 2) and initial concentrations of OCS and Br_2_ (by a factor of 3 and 2.5, respectively) do not affect either the slope of the straight line (*k*_1_/*k*_2_) or its intercept. This observation seems to indicate that the additional consumption of Br_2_ in secondary reactions is unlikely. The observed non-zero intercept in Figure 5 and Figure 6 could potentially be due to the presence of O atoms, which are known to be generated in microwave discharge of F_2_. Moreover, oxygen atoms react much faster with Br_2_ than with OCS.

O + Br_2_ → Br + BrO (R3)

*k*_3_ = 9.85 × 10^−16^ T^1.41^ exp(543/T) cm^3^ molecule^−1^ s^−1^ (T = 220–950 K) [5];

O + OCS → products (R4)

where *k*_4_ = 1.92 × 10^−12^ (T/298)^2.08^ exp(−1524/T) cm^3^ molecule^−1^ s^−1^ (T = 220–960 K) [6]. In addition, the contribution of small amounts of H atoms and OH radicals from the microwave discharge to the consumption of Br_2_ cannot be ruled out, considering that H and OH are more reactive towards Br_2_ than toward OCS.

To be more confident in the relative measurement of *k*_1_, we carried out another series of experiments in which the rate constant of Reaction (R1) was measured relative to that of the reaction of F atoms with Br_2_, but within the framework of a different approach (RRM2). The experiments consisted of rapid titration of F atoms in the reaction with a mixture of OCS and Br_2_ and measuring the yield of FBr as a function of the [OCS]/[Br_2_] ratio. The fraction of the initial concentration of F atoms converted into FBr in Reaction (R2) is equal to the following:

$$\left[FBr\right]=\frac{k_{2}\left[{Br}_{2}\right]}{k_{2}\left[{Br}_{2}\right]+k_{1}\left[OCS\right]+k_{w}}\times {\left[F\right]}_{0}$$

Rearranging this equation gives the following equation:

(2) $$\frac{{\left[F\right]}_{0}}{\left[\mathrm{FBr}\right]}-1=\frac{k_{1}\left[\mathrm{OCS}\right]}{k_{2}\left[{\mathrm{Br}}_{2}\right]}+\frac{k_{w}}{k_{2}{[\mathrm{Br}}_{2}]}$$

**Figure 7:**
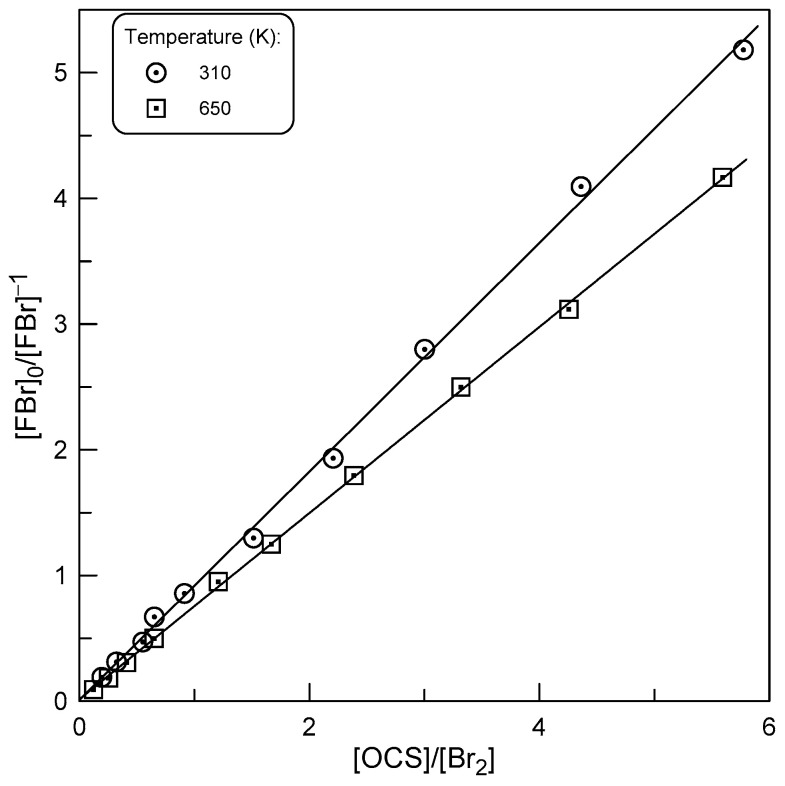
Yield of FBr upon titration of F atoms in reaction with OCS/Br_2_ mixtures at different temperatures.

At a constant Br_2_ concentration, the second term in Equation (2) is constant and *k*_1_/*k*_2_ can be determined as the slope of the linear relationship ([F]_0_/[FBr] − 1) versus [OCS]/[Br_2_] ratio. The concentration of FBr was monitored in the presence of Br_2_ and in the absence of OCS, which corresponds to [FBr]_0_ = [F]_0_, and in the presence of OCS in the reactor, which corresponds to the fraction of [F]_0_ that reacted with Br_2_. Examples of the observed experimental data are shown in Figure 7 and final values of *k*_1_ from these experiments are given in Table 1.

### 2.3. Rate Constant of Reaction (R1): Temperature Dependence

**Figure 8:**
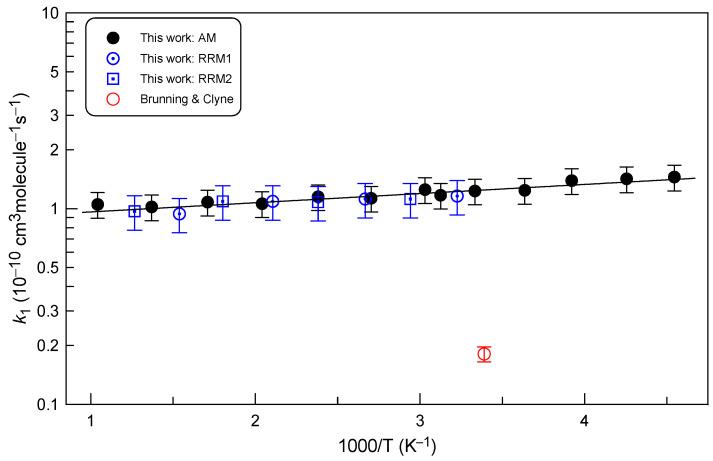
Summary of the measurements of the rate constant of Reaction (R1). Error bars on the present data correspond to 15% and 20% uncertainty of the absolute and relative measurements of *k*_1_, respectively. Red circle: Brunning and Clyne [3].

All the data available for the rate constant of Reaction (R1) are displayed in Figure 8. Concerning the data from the present study, we can note a very good agreement between the relative rate method, which does not require measurements of the absolute concentrations of the species, and the absolute measurements of the reaction rate constant. Negative temperature dependence observed for *k*_1_ can be described by the following Arrhenius equation:

*k*_1_ = (8.95 ± 0.52) × 10^−11^ exp((102 ± 20)/T) cm^3^ molecule^−1^ s^−1^

in the temperature range from 220 to 960 K and with 2σ uncertainties representing the precision of the fit.

Previously, Reaction (R1) has been investigated in only one study [3]. Brunning and Clyne [3], using a discharge-flow system combined with mass spectrometric detection of the species involved, reported *k*_1_ = (1.81 ± 0.16) × 10^−11^ cm^3^ molecule^−1^ s^−1^ at T = 295 K and 1 Torr total pressure. This value is lower by a factor of 7 than that determined in the present work. It is difficult for us to explain such a large discrepancy, especially since both studies were carried out in similar flow reactors and the reaction rate constant was determined using the similar experimental protocol, from the kinetics of OCS consumption in excess of F atoms. It seems that the main reason for the disagreement lies in the determination of the absolute concentrations of F atoms. In the study of Brunning and Clyne [3], F-atom concentrations were determined using the titration reaction F+ Cl_2_ → Cl + FCl. Unfortunately, the article does not provide details of the measurements and does not specify whether only the initial concentration of F atoms was measured or their kinetics along the reaction zone was controlled. In the present work, a similar procedure, the titration of F atoms with excess Br_2_, was applied for measurements of their absolute concentrations and [F] in the reactor was monitored in situ as a function of the reaction time. In addition, relative measurements of the rate constant, independent of the absolute concentration measurements, support the absolute data for *k*_1_.

### 2.4. Theoretical Findings

There are three different pathways for the title reaction, which are shown below:

OCS + F → SF + CO(R1a)

    → FO + CS (R1b)

      → OC(F)S (+M)(R1c)

**Figure 9:**
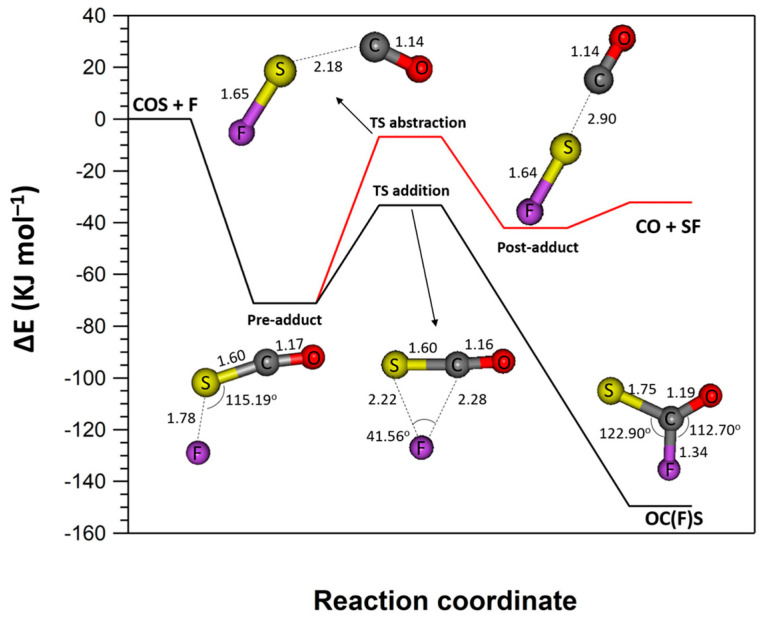
Profile of the potential energy surface for the reaction of F atoms with OCS. Structure optimizations and single-point energy calculations (in kJ mol^−1^), carried out using the quantum composite G3B3 method, are also presented. The red and black lines correspond to the S abstraction (R1a) and F-atom addition (R1c) pathways, respectively. The bond lengths are in Å.

Reaction (R1b) was calculated to be highly endothermic with energy of 461 kJ mol^−1^ using the G3//MP2/aug-cc-pVDZ. It is highly improbable that this channel will occur and thus it is not further discussed in the following section of the manuscript. On the contrary, pathways (R1a) and (R1c) are exothermic. The optimized geometries of reactants, products, TS, and intermediate species formed along the reaction coordinate with the G3B3 method are shown in Figure 9. The relative energies with the corresponding zero-point energies (ZPEs) for each conformation with the two different computational methods are given in Table 2. As displayed in Table 2, there is a relatively good agreement between the relative energies calculated with the two different methods. The reaction of F with OCS initially proceeds with the formation of a stable adduct (named pre-adduct in the following) where F atoms approach the OCS from the S site. The conformation of this adduct locked with G3B3 is displayed in Figure 9.

The relative energies calculated for the pre-adduct with G3B3 and G3//MP2/aug-cc-pVDZ were −71.3 kJ mol^−1^ and −67.7 kJ mol^−1^, respectively, corresponding to a relative stable conformation. We locked two different TS conformations corresponding to the abstraction (R1a) and addition (R1c) pathways, respectively. In both cases, submerged reaction barriers were calculated, indicating a possible inverse temperature dependence of the corresponding rate coefficient, in agreement with the experimental observations. Concerning the (R1a), channel the calculated energies were −6.9 kJ mol^−1^ and −2.0 kJ mol^−1^ with G3B3 and G3//MP2/aug-cc-pVDZ, respectively. The relative energy of the TS calculated for (R1c) was significantly lower, in the range of −33 kJ mol^−1^. A post-adduct was locked for the (R1a) channel with the G3B3 theory (but not with G3//MP2/aug-cc-pVDZ) with a calculated energy of −42.2 kJ mol^−1^ before the final products formation (i.e., CO and SF). Regarding channel (R1c), the final product was the OC(F)S radical, with a calculated energy of −149.6 and −146.6 kJ mol^−1^ with G3B3 and G3//MP2/aug-cc-pVDZ, respectively. It should be noted that a small basis set was used for the ab-initio theoretical calculations. While the use of more advanced basis sets would yield more accurate energy estimations, the objective of these calculations was to support the experimental data and provide a relative comparison of the two reaction pathways.

The intermediate energized adduct can dissociate back into reactants or to reaction products (Reaction (R1a)), or be collisionally stabilized (Reaction (R1c)). For the addition channel to take place (adduct stabilization), relatively high pressure is usually required to remove excess energy of the excited adduct via collisions with a third body (the bath gas in our experiments). It is clear that the competition between pathways (R1a) and (R1c) will be pressure and temperature dependent with a possible significant contribution of addition channel (R1c) at high pressures. However, under the experimental conditions of our measurements, at a total pressure of 2 Torr, a noticeable contribution to *k*_1_ (~10^−10^ cm^3^ molecule^−1^ s^−1^) of the addition channel seems very unlikely. For this reason, we did not consider it necessary to measure the reaction rate constant as a function of pressure. Using mass spectrometry, we detected the formation of the SF radical in Reaction (R1) directly at *m*/*z* = 51 (SF^+^) and upon its conversion to BrSF (*m*/*z* = 130/132, BrSF^+^) in reaction with Br_2_. Although the absolute concentrations of SF were not measured, judging by the relative mass spectrometric signals, the SF formation channel (1a) is the main, if not the only, reaction channel under the experimental conditions of the study. Reaction (R1) turns out to be a convenient and effective source of SF radicals, which opens up prospects and opportunities for detailed laboratory studies of elementary reactions involving SF.

## 3. Materials and Methods

### 3.1. Experimental Setup

The experimental setup, consisting of a discharge-flow reactor combined with a modulated molecular beam mass spectrometer with electron impact ionization, has been used extensively in the past [4,5,6,7,8]. Therefore, only a brief description will be presented herein. It contains the gas introduction vacuum lines, the flow reactor, and the differentially pumped stainless steel high-vacuum chamber, which houses the quadrupole mass spectrometer (Balzers, QMG 420, Balzers Aktiengesellschaft, Liechtenstein). The chemical composition of the reactive system was monitored by sampling the gas-phase molecules from the flow reactor and detecting them using the mass spectrometer. The gas mixture sampled from the flow reactor was modulated by a tuning-fork chopper (35 Hz), ionized through the impact with high kinetic energy electrons (30 eV) emitted by the ion source of the mass spectrometer and detected using an electron multiplier. Subsequently, the mass spectrometric signals were filtered and amplified with a lock-in amplifier and recorded for further analysis.

The reaction time in the flow reactor was determined by the position of the tip of the movable injector relative to the sampling cone of the mass spectrometer (Figure 1 and Figure 4) and by the linear flow velocity in the reactor, which was in the range (1720–2460) cm s^−1^.

All experiments were carried out at a total pressure of 2 Torr, using He as the carrier gas. To provide an extended temperature range for kinetic measurements, two flow reactors were used. The first one used at low temperatures (220–330 K) consisted of a Pyrex tube (45 cm length and 2.4 cm i.d.); temperature regulation was achieved by circulating thermostated ethanol (Figure 1). The second flow reactor (T = 310–960 K) consisted of an electrically heated quartz tube (45 cm length and 2.5 cm i.d.) with water-cooled attachments (Figure 4) [8].

Fluorine atoms were generated from the microwave discharge in trace amounts of F_2_ in He in a ceramic (Al_2_O_3_) tube (Figure 1 and Figure 4), which was used to avoid potential reactions of F atoms with the glass surface inside the microwave cavity. F atoms were detected at *m*/*z* = 98/100 (FBr^+^) after being converted to FBr prior to their sampling by Reaction (R2) with excess Br_2_ (Figure 1).

The absolute concentrations of F atoms were determined through their titration with excess Br_2_. The measurement procedure consisted of the chemical conversion of F atoms into FBr upon their complete consumption in Reaction (R2), which relates the initial concentration of F atoms to the consumed fraction of [Br_2_] and the formed concentration of FBr: [F]_0_ = [FBr] = Δ[Br_2_]. The absolute concentrations of OCS and Br_2_ in the reactor were calculated from their flow rates obtained from pressure drop measurements in their mixtures in He stored in calibrated volumes.

The purities of the gases used were as follows: He > 99.9995% (Alphagaz, Air Liquide France Industrie, Paris, France), passed through liquid nitrogen trap; Br_2_ > 99.99% (Aldrich, St. Louis, MO, USA); F_2_, 5% in helium (Alphagaz), OCS > 99.99% (15% in He, Messer, Bad Soden, Germany).

### 3.2. Computational Methodology

All electronic structure calculations were carried out using the Gaussian 16 program suite [9]. The calculations were performed using two different methods. The first one concerned the quantum composite G3B3 method [10], which is a variation of G3 theory [11]. This compound method consists of geometry optimization and vibrational frequency calculations at the B3LYP/6-31G(d) [12] level of theory. Thereafter, a series of single-point energy calculations at four different higher levels of theory QCISD(T)/6-31G(d), MP4/6-31+G(d), MP4/6-31G(2df,p), and MP2/G3Large were performed using the optimized structure. Calculations were also carried out with the G3//MP2/aug-cc-pVDZ method. More precisely, all geometry optimization and vibrational frequency calculations were conducted at the MP2 [13]/aug-cc-pVDZ [14] level of theory. Subsequently, single-point energy calculations were realized for the optimized structures, manually applying the G3 theory and scaling the harmonic frequencies by a factor of 0.959 as adopted from the CCCBDB database [15]. Further improvements in electronic energies were achieved by applying spin–orbit coupling corrections. In addition, the potential energy surfaces were computed by performing intrinsic reaction coordinate (IRC) calculations in order to verify that the located transition state (TS) structures are connected to the proper reactants and products.

## 4. Conclusions

In this study, a discharge-flow reactor combined with an electron impact ionization mass spectrometer was employed to study the reaction of fluorine atoms with carbonyl sulfide. The reaction rate constant was determined at a total pressure of 2 Torr as a function of temperature in a wide temperature range (T = 220–960 K) using an absolute and two different relative rate methods. The rate constant data obtained by the three methods are in good agreement with each other and provide the following Arrhenius expression: *k*_1_ = (8.95 ± 0.52) × 10^−11^ exp((102 ± 20)/T) cm^3^ molecule^−1^ s^−1^ (with an estimated total uncertainty of 15% independent of temperature), with a *k*_1_ value that is a factor of 7 higher compared to the only previous measurement at room temperature. The theoretical calculations performed within this study support the negative sign of the activation energy. The SF radical was observed as the reaction product. The experimental observations suggest that the SF-forming channel, F + OCS → SF + CO, is the main, if not the only, reaction channel under the experimental conditions of the study.

## Figures and Tables

**Table 1:** Summary of the measurements of the rate constant of Reaction (R1).

*T* (K)	*k*_1_ ^a^	Method ^b^	Reactor Surface ^c^
220	1.45	AM	HW
235	1.42	AM	HW
255	1.39	AM	HW
275	1.24	AM	HW
300	1.23	AM	HW
310	1.16	RRM2	Q
320	1.17	AM	Q
330	1.25	AM	HW
340	1.12	RRM1	Q
370	1.13	AM	Q
375	1.12	RRM2	Q
420	1.15	AM	Q
420	1.08	RRM1	Q
475	1.09	RRM2	Q
490	1.06	AM	Q
555	1.09	RRM1	Q
585	1.08	AM	Q
650	0.94	RRM2	Q
730	1.02	AM	Q
790	0.97	RRM1	Q
960	1.05	AM	Q

^a^ units of 10^−10^ cm^3^ molecule^−1^ s^−1^, statistical 2σ uncertainty is (2–3)%, total estimated uncertainty on *k*_1_ is 15% and 20% for absolute and relative measurements, respectively; ^b^ AM: absolute measurements; RRM1 and RRM2: two relative rate methods (see text); ^c^ HW: halocarbon wax; Q: quartz.

**Table 2:** Calculated energies (kJ mol^−1^) for the title reaction using the G3B3 and G3//MP2/aug-cc-pVDZ methods. Spin coupling corrections for F and SF were applied.

Method	Pre-Adduct	TS	Post-Adduct	Final Products
OCS +F → SF + CO (R1a)				
G3B3	−71.3	−6.9	−42.2	−32.2
G3//MP2/aug-cc-pVDZ	−67.7	−2.0		−27.8
OCS +F → OC(F)S (R1c)				
G3B3	−71.3	−33.3		−149.6
G3//MP2/aug-cc-pVDZ	−67.7	−36.4		−146.6

## Data Availability

The data supporting reported results are available in this article.
